# Application of Radiomics Analysis Based on CT Combined With Machine Learning in Diagnostic of Pancreatic Neuroendocrine Tumors Patient’s Pathological Grades

**DOI:** 10.3389/fonc.2020.521831

**Published:** 2021-02-11

**Authors:** Tao Zhang, YueHua Zhang, Xinglong Liu, Hanyue Xu, Chaoyue Chen, Xuan Zhou, Yichun Liu, Xuelei Ma

**Affiliations:** ^1^ Department of Biotherapy, Cancer Center, West China Hospital, Sichuan University, Chengdu, China; ^2^ State Key Laboratory of Biotherapy, West China Hospital, Sichuan University, Chengdu, China; ^3^ West China School of Public Health and West China Fourth Hospital, Sichuan University, Chengdu, China; ^4^ West China School of Medicine, West China Hospital, Sichuan University, Chengdu, China; ^5^ Department of Neurosurgery, West China Hospital, Sichuan University, Chengdu, China

**Keywords:** CT, pancreatic neuroendocrine tumors, texture analysis, pathological grading, radiomics, prediction model

## Abstract

**Purpose:**

To evaluate the value of multiple machine learning methods in classifying pathological grades (G1,G2, and G3), and to provide the best machine learning method for the identification of pathological grades of pancreatic neuroendocrine tumors (PNETs) based on radiomics.

**Materials and Methods:**

A retrospective study was conducted on 82 patients with Pancreatic Neuroendocrine tumors. All patients had definite pathological diagnosis and grading results. Using Lifex software to extract the radiomics features from CT images manually. The sensitivity, specificity, area under the curve (AUC) and accuracy were used to evaluate the performance of the classification model.

**Result:**

Our analysis shows that the CT based radiomics features combined with multi algorithm machine learning method has a strong ability to identify the pathological grades of pancreatic neuroendocrine tumors. DC + AdaBoost, DC + GBDT, and Xgboost+RF were very valuable for the differential diagnosis of three pathological grades of PNET. They showed a strong ability to identify the pathological grade of pancreatic neuroendocrine tumors. The validation set AUC of DC + AdaBoost is 0.82 (G1 vs G2), 0.70 (G2 vs G3), and 0.85 (G1 vs G3), respectively.

**Conclusion:**

In conclusion, based on enhanced CT radiomics features could differentiate between different pathological grades of pancreatic neuroendocrine tumors. Feature selection method Distance Correlation + classifier method Adaptive Boosting show a good application prospect.

## Introduction

Pancreatic neuroendocrine tumors (PNETs) are tumors that originate from the neuroendocrine system of the pancreas, accounting for 2%–10% of pancreatic tumors (1, 2). In recent years, researches on pancreatic neuroendocrine tumors have received more and more attention. According to the World Health Organization’s classification system, PNETs are classified into three pathological grades according to mitotic count and Ki-67 index: G1, G2, G3. In PNETs, G1 grade tumors have a lower degree of malignancy, and G2 and G3 tumors have a higher degree of malignancy (3, 4). Different treatment options for different grades of tumors are also different. At present, the more accepted treatment is surgical resection. For the unresectable or metastatic PNETs, local treatment, chemotherapy and targeted therapy can be used as treatment options (5–7). Of course, tumors with different malignant grades will also have an impact on the development of treatment options.

At present, enhanced CT examination has been used as a common imaging examination method for pancreatic tumors, and it is an important auxiliary tool for clinicians in evaluating tumor staging (8–10). However, enhanced CT is not able to directly determine the malignant grade of PNETs. The confirmed diagnosis still needs to rely on pathological diagnosis, which invisibly increases the difficulty of diagnosis and the suffering of patients. New methods are explored to identify the grade of carcinoma non-invasively by using the image data with the development of medical imaging and post-processing. It is valid to predict Gleason Score of Prostate cancer, malignancy stage of Colorectal cancer by analyzing image feature (11, 12). This suggests that radiomics information can predict pathological information to a certain extent. Compared with pathological information, there is a huge amount of valuable information hidden in the image. Notably, as an emerging non-invasive way, radiomic analysis makes a transformation of medical images into available data combined with other clinical information of patients, playing a crucial role in diagnosis of multiple tumors (13).

Texture analysis, one type of radiomics analysis, is a method of quantifying texture parameters by post-processing conventional images, mathematically analyzing and calculating the intensity and spatial distribution characteristics of image pixels (14). The main image sources are CT, MRI, PET, and some post-processing images. Enhanced CT texture analysis can reflect the uneven distribution of contrast agent inside and outside the blood vessel (15). In recent years, CT texture analysis also has been applied to the diagnosis, grading, and prognostic evaluation of various tumors, such as colorectal cancer (16–18).

It has been reported that preoperative texture analysis of PNETs patients provides a novel and feasible method for the diagnosis and prognosis of PNETs patients (19–23). However, as far as we know, although some studies have found that several texture parameters have potential application value in PNETs diagnosis and grading, the explored parameters are still incomplete and need further investigation. To show the full picture of the ability of texture parameters based on enhanced CT to differentiate pathological grading of PNETs, we conducted this retrospective study. This is the first time that five feature selection methods and nine classifiers have been used to help identify pancreatic neuroendocrine tumors with different pathological grades. The purpose of this study was to evaluate the ability of CT based radiomic combined with machine learning to identify pathological grade of pancreatic neuroendocrine tumor, and to compare the performance of five feature selection methods and nine classifiers.

## Materials and Methods

### Study Population

We retrospectively reviewed a computer database of PNETs patients treated at our hospital from March 2011 to November 2019, yielded 201 patients who had treated for PNETs with their clinical records and CT images. Clinical data including age, sex, location, tumor size, pathological typing, date of baseline CT, condition of secretory function and dates of surgery were recorded in our computer medical system. All CT images were exported through the hospital’s PACS (Picture Archiving and Communication System). One hundred nineteen patients were excluded after the initial evaluation on images and patient profiles, the reasons are as follows: Patients who did not undergo an enhanced abdominal CT scan within 2 months prior to surgery (n=38); The patient did not had a definite pathological results (n=36); Relevant tumor treatment history in other hospitals (n=41); the image quality does not meet the requirements (n=4). A total number of 82 patients were introduced in our study, finally. This study was approved by the Ethics Administration Office of the West China Hospital, Sichuan University, and the requirement for informed consent was waived.

### CT Acquisition

Before the treatment, all patients underwent contrast-enhanced abdomen CT examinations by a single 64-detector row scanner (Brilliance 64, Philips Medical Systems, Eindhoven, the Netherlands) with the following uniform scan parameters: beam pitch, 0.891; tube voltage,120 kVp; tube current, 200 mAs; detector collimation, 0.75 mm; slice thickness, 1.0 mm; reconstruction increment, 5.0 mm; rotation time, 0.42 s; and matrix, 512 x 512.

Images were obtained after intravenous administration of contrast agent (iohexol, 300 mg iodine/ml; Bayer Schering Pharma AG, Leverkusen, Germany) dosed to weight (1.5 ml/kg) at a rate of 2.5–3.0 ml/s through a power injector (Stellant D Dual Syringe, Medrad, Indianola, PA, USA). Computed tomography scanning was performed with 30–35 s for arterial-phase and 60–70 s for portal venous phase (24–26).

### Texture Features Extraction

We retrieved and extracted the digital imaging and communication medical data (400-bit gray scale) of the enhanced CT of the study patient from the image archiving system. In order to quantify the lesion segmentation and automatic quality characteristics, these data were loaded into a personal computer-based partial image feature extraction software (LIFEx v3.74, CEA-SHFJ, Orsay, France) for segmentation and texture analysis. In enhanced CT fusion images, regions of interest (ROIs) were drawn by hand around tumor lesions (27, 28). All CT data were selected for arterial phase. The drawing process of ROIs was done independently by two radiologists. The radiogist contoured along the tumor tissue slice by slice to draw the region of interest (ROI), and the three dimensional texture features were automatically generated with default setting (29).

The cystic, calcified and vascular shadows of the tumors were removed during the process. To ensure objectivity, we implemented mutual blindness for two radiologists. A third radiologist evaluated the ROIs sketched by the previous two radiologists and selected the more accurate ROIs to generate texture features. Texture features of all image data are then automatically calculated and extracted by computer software Lifex. A total of 40 subdivided texture features were extracted, including features from the first order (minimum value, maximum value, average value and standard deviation value, histogram-based matrix and shape-based matrix) and features from second or higher order [gray-level co-occurrence matrix (GLCM), gray-level zone length matrix (GLZLM), neighborhood gray-level dependence matrix (NGLDM), and gray-level run length matrix (GLRLM)].

### Machine Learning

The establishment of machine learning model includes two key points: feature selection by algorithm and modeling. The patients were randomly separated into two sets in the ratio of 3:1 as the training set and the validation set. Considering that there are so many features, over fitting will occur, which will affect the prediction performance of the model (30).

The purpose of feature selection is to reduce the effect of overfitting. Considering that there are many different selection methods at present, we evaluated five selection methods: Distance Correlation(DC), Random Forest (RF), Least Absolute Shrinkage And Selection Operator (LASSO), Extreme Gradient Boosting (Xgboost) and Gradient Boosting Decision Tree (GBDT). We apply all radiomics features selected to the classification algorithm to establish the discrimination model of different algorithm combinations for pathological grading of PNETs. The nine machine learning classifiers were: linear discriminant analysis (LDA), Support Vector Machines (SVM), Random Forest (RF), Adaptive Boosting (AdaBoost), K-nearest neighborhood (KNN), Gaussian Naive Bayes (GaussianNB), Logistic Regression (LR), Gradient Boosting Decision Tree(GBDT) and Decision Tree (DT). For each model, we repeated 10 times machine learning process to obtain the real distribution of classification. In analysis of diagnostic grading, we made receiver operating characteristic (ROC) curves of every diagnostic models. The discriminating power of the model was measured by the area under the curve (AUC) of the ROC curve. The predicted targets were pathological grade(G1, G2, and G3). Sensitivity is defined as the proportion of positive samples judged to be positive. Specificity is defined as the proportion of negative samples judged to be negative. The accuracy was defined as the percentage of the sum of true positive and true negative in the number of subjects. The association between texture parameters was evaluated using Pearson correlation coefficient test.

**Figure 1:**
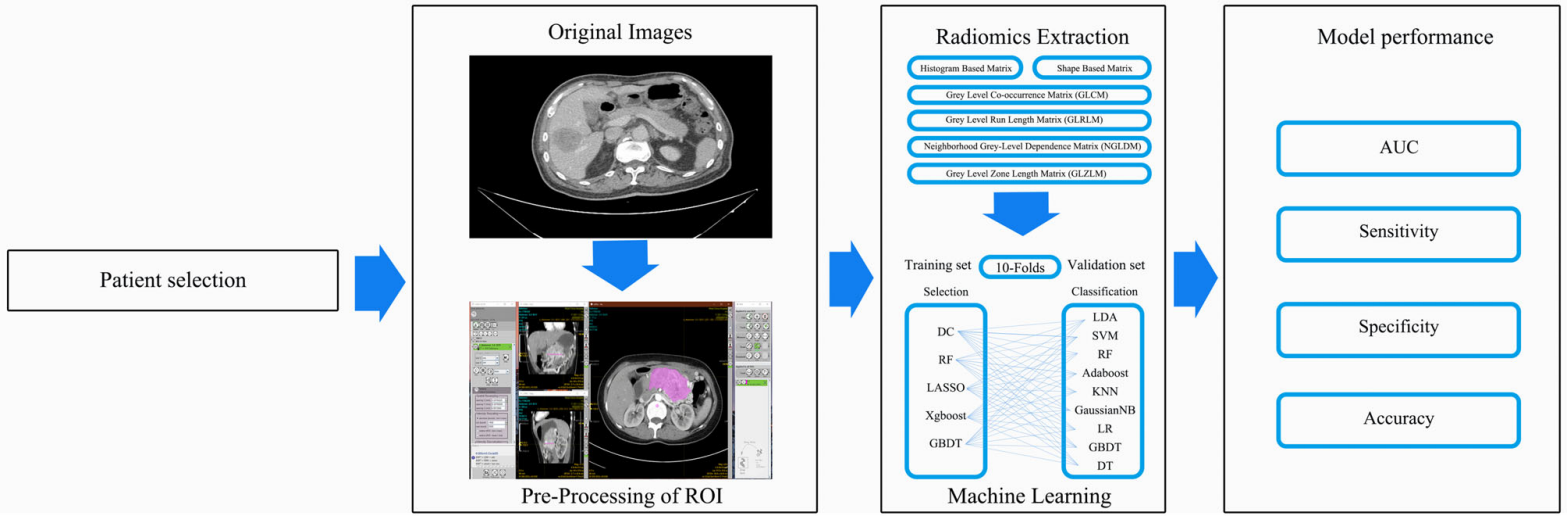
Study-process diagram.

A P value < 0.05 was considered to indicate statistical significance and all P values were based on two-sided testing. All regular statistical analyses were performed using the SPSS software (Version 20.0, IBM Corporation, Armonk, NY, USA). The machine learning algorithms were programmed using were performed on Python software (sklearn package). The study-process diagram is shown in **Figure 1**.

## Result

### Patient Population

Among the 82 patients (mean age, 52.6 years old; range: 24–77 years old) included in the present study, 52 patients were male and 30 patients were female. The pathological type of all patients was PNET. The median OS for this cohort was 58.2 months. As for the pathological results, there are 20 patients confirmed as G1, 33 patients were G2, and 29 patients were G3. There were 21 patients with tumor secretory function. Seven patients died and the remaining 75 patients survived.

We found that the gender, age and survival status of the patients had no significant relationship with the pathological grade of the tumor. (P > 0.05). At the same time, tumor location, tumor secretory function, vascular invasion, peripancreatic permeation, pancreatic duct dilatation, boundary form, calcification, pancreatic atrophy, and the maximum diameter of the tumor are not related to the pathological grade of the tumor.

The baseline characteristics of all patients and lesions were summarized in **Table 1**.

**Table 1:** Characteristics of patients and lesions.

	Low-grade	High-grade		Total
	G1	G2	G3	
No. of patients	20	33	29	82
Patient sex, no. (%)				
Male	13(65.0%)	20(60.6%)	19(65.5%)	52(63.4%)
Female	7(35.0%)	13(39.4%)	10(34.5%)	30(36.6%)
Age(range)				
Mean	49.2(28-70)	53.0(29-75)	54.6(24-77)	52.6(24-77)
Location, no. (%)				
Head	8(40.0%)	14(42.4%)	20(69.0%)	42(51.2%)
Neck Body Tail	4(20.0%) 3(15.0%) 5(25.0%)	8(24.2%) 5(15.2%) 6(18.2%)	2(6.9%) 3(10.3%) 4(13.8%)	14(17.1%) 11(13.4%) 15(18.3%)
Function, no. (%)				
Yes	2(10.0%)	10(30.3%)	9(31.0%)	21(25.6%)
No	18(90.0%)	23(69.7%)	20(69.0%)	61(74.4%)
Blood vessel is invasion, no. (%)				
Yes No	2(10.0%) 18(90.0%)	5(15.2%) 28(84.8%)	7(24.1%) 22(75.9%)	14(17.1%) 68(82.9%)
Peripancreatic permeation, no. (%)				
Yes No	0(0%) 20(100.0%)	4(12.1%) 29(87.9%)	4(13.8%) 25(86.2%)	8(9.8%) 74(90.2%)
Pancreatic duct dilatation, no. (%)				
Yes No	2(10.0%) 18(90.0%)	10(30.3%) 23(69.7%)	6(20.7%) 23(79.3%)	18(22.0%) 64(78.0%)
Border, no. (%)				
Clear Unclear	10(50.0%) 10(50.0%)	6(18.2%) 27(81.8%)	8(27.6%) 21(72.4%)	24(29.3%) 58(70.7%)
Calcification, no. (%)				
Yes No	2(50.0%) 18(50.0%)	3(9.1%) 30(90.9%)	6(20.7%) 23(79.3%)	11(13.4%) 71(86.6%)
Pancreatic atrophy, no. (%)				
Yes No	4(20.0%) 16(80.0%)	4(12.1%) 29(87.9%)	7(24.1%) 22(75.9%)	15(18.3%) 67(81.7%)
Maximum diameter(cm)	3.1(0.8–6.0)	4.9(1.0–12.0)	6.2(0.8–12.0)	4.9(0.8–12.0)
Survival Status, no. (%)				
Alive Dead	19(95.0%) 1(5.0%)	31(93.9%) 2(6.1%)	25(86.2%) 4(13.8%)	75(91.5%) 7(8.5%)
Median Overall Survival (month)	57.0	52.2	62.4	58.2

### Texture Features

**Figure 2:**
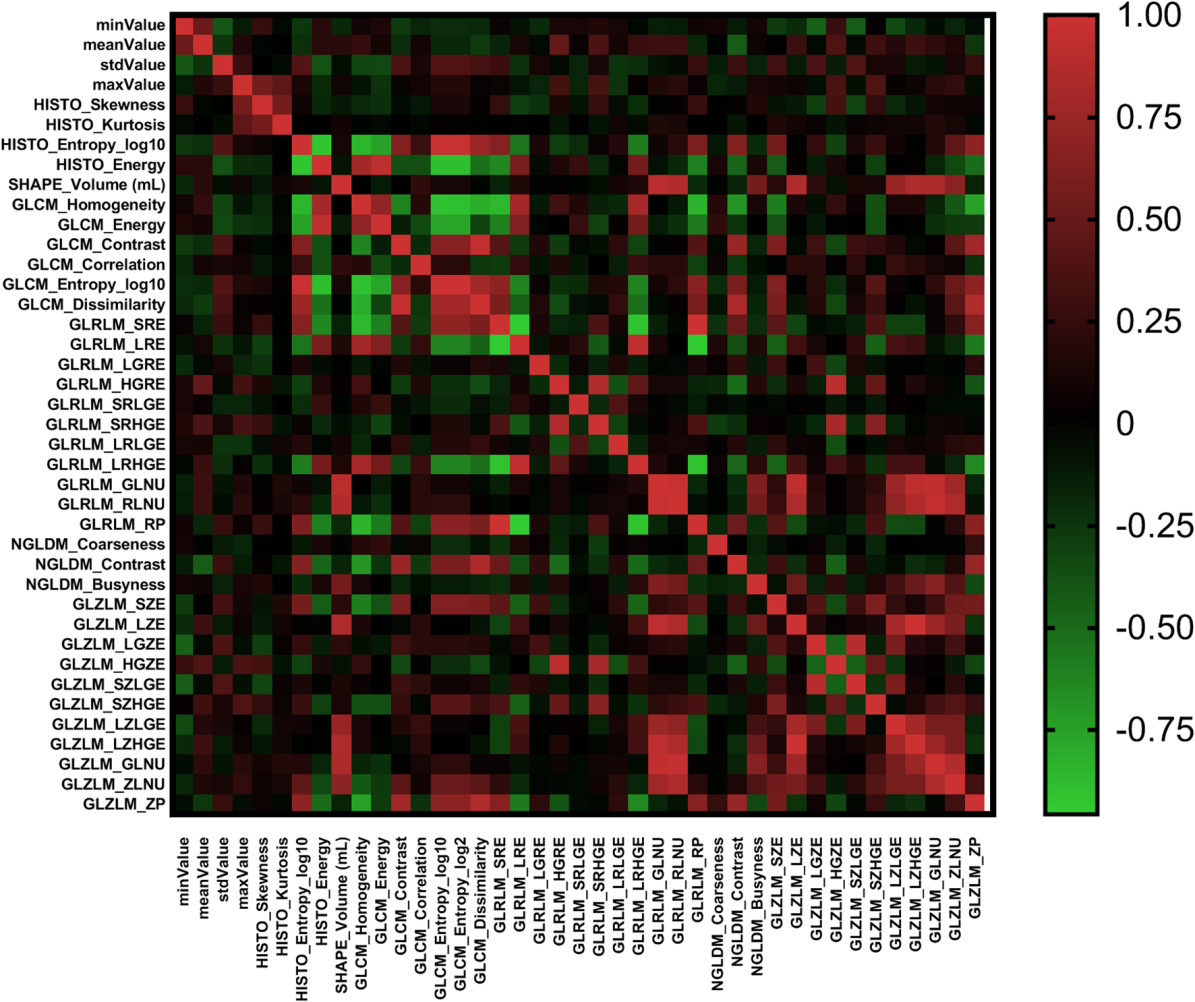
The heat map of Pearson correlation coefficients among radiomics features.

According to the Pearson correlation coefficient of the extracted features, most texture features were independent or weakly correlated. There were also a few features showing strong positive correlation and strong negative correlation. The correlation of all texture features was shown in **Figure 2** in the form of heatmap. In different comparisons, each selection method had selected different radiomics features. In terms of feature selection, RF and lasso select the most number of radiomics features, while Xgboost selects the least number of features. The feature selection results of each method were shown in **Table 2**.

**Table 2:** Selected features using Distance Correlation (DC), Random Forest (RF), Least Absolute Shrinkage and Selection Operator (LASSO), Extreme Gradient Boosting (Xgboost), and Gradient Boosting Decision Tree (GBDT).

Selection method	Selected features		
	G1 vs G2	G2 vs G3	G1 vs G3
DC	maxValue SHAPE_Volume (ml) GLCM_Correlation GLRLM_GLNU GLRLM_RLNU GLRLM_LRLGE GLZLM_ZLNU GLZLM_GLNU	HISTO_Kurtosis HISTO_Skewness SHAPE_Volume (ml) GLRLM_LGRE GLRLM_SRHGE GLRLM_LRLGE GLRLM_HGRE GLZLM_HGZE	stdValue maxValue HISTO_Skewness GLCM_Correlation GLRLM_HGRE GLRLM_SRHGE GLZLM_SZLGE GLZLM_HGZE
RF	minValue maxValue HISTO_Skewness SHAPE_Volume (ml) GLCM_Homogeneity GLCM_Contrast GLCM_Correlation GLCM_Entropy_log10 GLRLM_LGRE GLRLM_LRLGE GLRLM_LRHGE GLRLM_GLNU GLRLM_RLNU GLZLM_LZE GLZLM_LGZE GLZLM_LGZE GLZLM_SZLGE GLZLM_LZHGE	meanValue maxValue HISTO_Skewness HISTO_Kurtosis SHAPE_Volume (ml) GLCM_Contrast GLRLM_LRE GLRLM_LGRE GLRLM_HGRE GLRLM_LRLGE GLRLM_GLNU GLRLM_RP NGLDM_Coarseness GLZLM_LZE GLZLM_HGZE GLZLM_SZLGE GLZLM_SZHGE GLZLM_LZHG GLZLM_ZLNU	meanValue stdValue maxValue HISTO_Skewness GLCM_Homogeneity GLCM_Entropy_log10 GLRLM_SRE GLRLM_SRLGE GLRLM_SRHGE GLRLM_LRLGE GLRLM_LRHGE GLRLM_RLNU GLRLM_RP NGLDM_Contrast GLZLM_LZE GLZLM_LGZE GLZLM_HGZE GLZLM_LZHGE
LASSO	minValue maxValue HISTO_Kurtosis SHAPE_Volume (ml) GLRLM_HGRE GLRLM_SRHGE GLRLM_LRHGE GLRLM_GLNU GLRLM_RLNU GLZLM_LZE GLZLM_HGZE GLZLM_SZHGE GLZLM_LZHGE GLZLM_ZLNU	minValue meanValue stdValue maxValue SHAPE_Volume (ml) GLRLM_HGRE GLRLM_SRHGE GLRLM_LRHGE GLRLM_GLNU GLRLM_RLNU GLZLM_LZE GLZLM_HGZE GLZLM_SZHGE GLZLM_LZHGE GLZLM_GLNU GLZLM_ZLNU	minValue meanValue maxValue HISTO_Kurtosis GLRLM_HGRE GLRLM_LRHGE GLRLM_GLNU GLRLM_RLNU GLZLM_LZE GLZLM_HGZE GLZLM_SZHGE GLZLM_LZHGE GLZLM_GLNU GLZLM_ZLNU
Xgboost	maxValue GLCM_Correlation GLZLM_LGZE GLZLM_SZLGE GLZLM_ZP	minValue GLRLM_SRE GLRLM_LRLGE NGLDM_Coarseness GLZLM_LZE GLZLM_ZP	meanValue maxValue GLZLM_LGZE GLZLM_SZLGE
GBDT	minValue maxValue HISTO_Kurtosis SHAPE_Volume (ml) GLCM_Correlation GLRLM_RLNU NGLDM_Contrast NGLDM_Busyness GLZLM_LGZE GLZLM_SZLGE GLZLM_ZP	minValue stdValue maxValue HISTO_Skewness HISTO_Kurtosis SHAPE_Volume (ml) GLCM_Correlation GLRLM_LGRE GLRLM_SRLGE GLRLM_LRLGE GLRLM_RLNU NGLDM_Coarseness NGLDM_Contrast GLZLM_HGZE GLZLM_SZHGE GLZLM_GLNU	meanValue maxValue GLCM_Correlation NGLDM_Contrast GLZLM_HGZE GLZLM_SZLGE GLZLM_LZHGE

GLCM, Gray-Level Co-Occurrence Matrix; GLRLM, Gray Level Run Length Matrix; SRE, Short Run Emphasis; LRE, Long Run Emphasis; LGRE, Low Grey Level Run Emphasis; HGRE, High Gray Level Run Emphasis; SRLGE, Short-Run Low Grey Level Emphasis; SRHGE, Short-Run High Grey Level Emphasis; LRLGE, Long-Run Low Grey Level Emphasis; LRHGE, Long-Run High Grey Level Emphasis; GLNU, Grey Level Non-Uniformity; RLNU, Run Length Non-Uniformity; RP, Run Percentage; NGLDM, Neighborhood Grey Level Difference Matrix; GLZLM, Gray Level Zone Length Matrix; SZE, Short Zone Emphasis; LZE, Long Zone Emphasis; LGZE, Low Gray Level Zone Emphasis; HGZE, High Grey Level Zone Emphasis; SZLGE, Short Zone Low Grey Level Emphasis; SZHGE, Short Zone High Grey Level Emphasis; LZLGE, Long Zone Low Grey Level Emphasis; LZHGE, Long Zone High Grey Level Emphasis; ZLNU, Zone Length Non-Uniformity; ZP, Zone Percentage.

### Model Performance

We made three comparisons: G1 vs G2, G2 vs G3, and G1 vs G3. Each comparison established 45 diagnostic models and the best diagnostic model of each comparison was to select the one of the highest validation set AUC values in all models and in the three comparisons, there was no over-fitting or under-fitting in the model constructed by the algorithm combination. And once the diagnosis performance of the model appears overfitting or underfitting, the combination of algorithms used in the model was not considered to be the best model.

**Figure 3:**
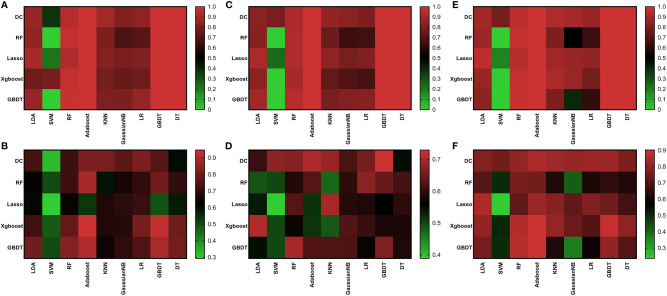
Heat map of AUC results of all algorithms. Training set of G1 vs G2 **(A)**; Validation set of G1 vs G2 **(B)**; Training set of G2 vs G3 **(C)**; Validation set of G2 vs G3 **(D)**; Training set of G1 vs G3 **(E)**; Validation set of G1 vs G3 **(F)**.

About the comparison of G1 and G2, the AUC values of most models were between 0.60 and 0.82. There was also a few model with high AUC values in comparison with G1 and G2, but showed overfitting in G2 and G3 comparisons (AUC of validation set is much lower than AUC of training set). Like RF + AdaBoost, Xgboost + AdaBoost, Xgboost + GBDT, GBDT + RF, GBDT + AdaBoost, and GBDT + GBDT, although they had high AUC values in the comparison of G1 and G2, they all showed over-fitting in the comparison of G2 and G3 (**Figure 3**). These models were not included in the selection of the best model. The highest value was 0.82 observed in DC+AdaBoost. In the validation set, the sensitivity, specificity, accuracy, and AUC of the model were 0.65, 0.84, 0.82, and 0.75. For the comparison of G2 and G3, the AUC values of most models were between 0.50 and 0.73. The highest value was 0.73 observed in DC+GBDT. In the validation set, the sensitivity, specificity, accuracy, and AUC of the model were 0.75, 0.64, 0.73, and 0.68, respectively. For the comparison of G1 and G3, the AUC values of most models were between 0.62 and 0.86. The highest value was 0.73 observed in Xgboost+RF. For the model (Xgboost+RF) in the validation set, the sensitivity, specificity, accuracy, and AUC of the model were 0.65, 0.87, 0.86, and 0.78, respectively.

**Figure 4:**
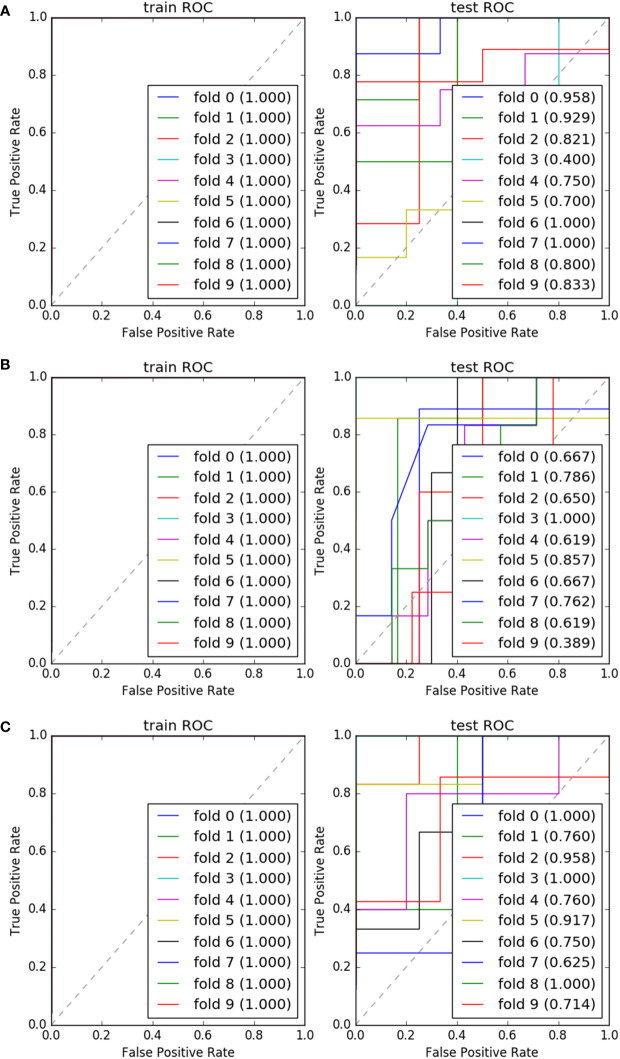
The ROC and AUC results of 10 fold of “DC+AdaBoost” in training set and validation set. G1 vs G2 **(A)**; G2 vs G3 **(B)**; G1 vs G3 **(C)**.

We found that the model constructed by the combination of DC + AdaBoost, DC + GBDT and Xgboost+RF was very valuable for the differential diagnosis of three pathological grades of PNET, and these models did not show over-fitting and under-fitting. The model performance of the combination of these three algorithms was shown in **Table 3**. DC + AdaBoost has the best performance in the three comparisons G1 vs G2, G2 vs G3, and G1 vs G3). The ROC curves of the DC + AdaBoost models was shown in **Figure 4**.

**Table 3:** Diagnostic performance of the optimal discriminative model in validation set.

Model	G1 vs G2			G2 vs G3			G1 vs G3		
	DC + AdaBoost	DC + GBDT	Xgboost+RF	DC + AdaBoost	DC + GBDT	Xgboost+RF	DC + AdaBoost	DC + GBDT	Xgboost+RF
**Sensitivity**	0.56	0.57	0.50	0.67	0.75	0.63	0.60	0.59	0.65
**Specificity**	0.84	0.76	0.84	0.66	0.64	0.60	0.88	0.86	0.87
**AUC**	0.82	0.75	0.78	0.70	0.73	0.57	0.85	0.82	0.86
**Accuracy**	0.75	0.71	0.72	0.65	0.68	0.61	0.77	0.76	0.78

The detailed AUC values for all models were shown in **Supplementary Material 1**.

## Discussion

In this study, we construct diagnostic models through cross combination of five feature selection methods and nine classification methods based on radiomics features. We evaluated the ability of the model to identify the pathological grade of PNETs, and explored the potential application value of machine learning combined with radiomics in the diagnosis of PNETs.

One of the main findings of this study was that the model constructed by feature selection method Distance Correlation + classifier method Adaptive Boosting had a strong ability to predict the pathological grade of PNETs. The validation set AUC of DC + AdaBoost is 0.82 (G1 vs G2), 0.70 (G2 vs G3), and 0.85 (G1 vs G3), respectively. As the result shows, these models had satisfactory results to indicate the pathological grade of PNETs (G1,G2, and G3). Models of DC + GBDT and Xgboost + RF also showed good diagnostic performance.

At present, CT has been widely used in the diagnosis, monitoring and prognosis evaluation of pancreatic neuroendocrine tumors. Enhanced CT can further differentiate tumors from normal tissues by using contrast media, especially for the detection of vascular proliferation and small lesions. It can improve the accuracy of clinical staging of cancer patients and help formulate treatment strategies. Especially, enhanced CT is suitable for breast and abdominal tumors and has certain diagnostic advantages (31–33). Texture analysis uses mathematically defined parameters to estimate the distribution of gray scale, roughness, and regularity within the lesion. So we can quantify some of the image features of the tumor by analyzing these parameters. The significant role of texture analysis in diagnosis and prognosis in combination with ultrasound, CT, MRI, and PET/CT has been confirmed. In some previous studies, it has been reported the value of texture analysis for the diagnosis and prognosis of various types of cancers, including lung, stomach, breast and rectal tumor (34–37). Heterogeneity is recognized as a characteristic of malignant tumors (38). Heterogeneity may be related to gene changes, tumor microenvironment and other factors that are different from normal tissues. Previous studies have also confirmed that CT texture features can also reflect the microenvironment of tumor vessels (39).

A negative finding is that indicators such as pancreatic duct dilatation, peripancreatic infiltration, vascular invasion, pancreatic atrophy and clear pancreatic boundaries do not indicate the pathological grading of tumors. It’s contrary to some previous studies (20, 21, 40). We do not deny that because of its higher malignancy. High-grade PNETs should grow faster and be more invasive than low-grade PNETs. Although many studies have found some correlation between pathological grade and imaging features of PNETs, there is not enough clear evidence that the morphology of tumors, like pancreatic duct dilatation, vascular invasion and other factors can clearly indicate the pathological grade of PNETs. However, previous studies have found that G1/G2 patients have clearer tumor boundaries than G3 patients. Larger tumor diameter, pancreatic duct dilatation, vascular invasion and other manifestations were more common in G2/G3 patients, and had statistical correlation (19, 41). This may be due to the limited sample size of this study, and more evidence from similar studies is needed to support these views. At present, pathological grading of PNETs still requires pathological sections to determine its grading. It is difficult but necessary to find the best radiomics feature of machine learning algorithm.

Appropriate selection method plays an important role in the performance of classifier. Previous studies on radiomics used many methods for feature selection, such as Mann Whitney U test with AUC of ROC, random forest and student’s t-test with recursive feature extraction, etc (42, 43). For our research, the number of extracted features is large, which increases the chance to select the optimal feature, but also increases the difficulty of selection. We consider using five different artificial intelligence methods for feature selection, which is better than using a single selection method in previous studies. In fact, three feature selection methods, Lasso, DC, and GBDT have been used in previous studies (44). On this basis, we add RF and Xgboost methods. The algorithm used in Xgboost is the improvement of GBDT, which can be used for classification and regression problems (45).

From our feature selection results, we can find that the features selected by different selection algorithms are not identical. Some algorithms select a lot features, such as LASSO, RF, and some algorithms select few features, such as Xgboost, but some features can be selected by most algorithms. Maxvalue is a feature describing the maximum value of a tumor image. This is a parameter based on the overall evaluation. In theory, high-grade tumors have more angiogenesis, and their overall characteristics are relatively more complex, which partly explains why maxvalue can describe high-grade PNETs features (23). In fact, we also find that maxvalue is chosen by almost all the selection algorithms. Many previous studies have found that the pathological grading of PNETs is closely related to the parameters of HISTO (Skewness, Kurtosis, Entropy and Energy) (21, 23, 40, 46). However, in our study, only skewness and kurtosis are selected by algorithms, indicating that they are related to pathological grade. We also found SHAPE_Volume (# ml), which is the same first-order parameter, has an indicative value for the pathological grading of PENTs. SHAPE_Volume (# ml) is the Volume of Interest. This reflects the shape characteristics of malignant tumors. The grey level co-occurrence matrix (GLCM) takes into account the arrangements of pairs of voxels to calculate textural indices. GLCM_Correlation is the linear dependency of grey-levels in GLCM. The grey-level run length matrix (GLRLM) gives the size of homogeneous runs for each grey level. GLRLM_RLNU is the length of the homogeneous runs. GLZLM-ZLNU is the length of the homogeneous zones. The neighborhood grey-level different matrix (NGLDM) corresponds to the difference of grey-level between one voxel and its 26 neighbors in three dimensions (eight in 2D). NGLDM_Coarseness is the level of spatial rate of change in intensity (27). These parameters reflect the differences in gray scale and voxel manifestations of tumors with different pathological grades. The imaging manifestations of malignant tumors are analyzed in a more detailed way.

In addition to finding the best diagnostic model, we also found that some of the models performed poorly. A previous study used LDA and SVM classifier machine learning methods to identify glioblastoma (GBM) and anaplastic oligodendrocytoma (AO), and found that the AUC of testing set was all above 0.90 (47). However, in our study, these two classification algorithms do not show good diagnostic performance. In particular, the models using SVM algorithm often show over-fitting or under-fitting. We find that the models using the rest of the classification algorithms perform better than all SVM based models, and the improvement of the models using different selection algorithms is limited. SVM algorithm is usually used to solve machine learning problems with small samples. This seems to be a good fit for the small sample size of this study, but in fact it shows a disappointing diagnostic performance. Compared with SVM, AdaBoost algorithm is a modified boosting algorithm, which can adaptively adjust the errors of classifiers. Through continuous training, AdaBoost can improve the ability of data classification. AdaBoost has low generalization error rate and can be applied to most classifiers.

Our research uses three-dimensional texture analysis, which can provide more information than the two-dimensional analysis. Compared with previous studies, based on image parameters and imaging parameters, this study introduces more comprehensive parameters such as GLCM, GLRLM, and GLZLM. Many previous studies only studied some parameters of HISTO and morphological characteristics of tumors. We use almost all the machine learning methods involved in the current research to analyze. This can intuitively compare the performance of various algorithm combinations and indicate the best combination. Another advantage of our study is that we have complete preoperative imaging, clinical and pathological data for reference. And the software used to extract texture parameters and execute machine learning to build prediction models in this study is free and open, which is conducive to replicate our research for other researchers.

There are still some shortcomings in this study. First, retrospective design may lead to selection bias. Then the sample size of this study is small and only included in patients undergoing abdominal enhanced CT examination, and the number of pathological grades is different, which may have certain selection bias. Future research needs a larger sample size to evaluate the application value of machine learning and radiomics in describing tumor pathological grading. Secondly, we only roughly defined the time points for performing enhanced CT examination before treatment, which resulted in different time points for enhanced CT examination, which led to deviations in the evaluation of texture features. Then, only the texture features extracted from the arterial phase CT images are used to establish the prediction model, while CT images of other phases are not explored. Finally, due to the lack of external validation, we cannot ensure that our model will have the same diagnostic performance when dealing with external data sets.

## Conclusion

The preoperative enhanced CT image texture analysis to predict the pathological grade of PNETs patients has a potential application. Radiomics analysis is expected to assist radiologists in obtaining more information from images.

## Data Availability Statement

The dataset generated for this study can be obtained from the correspondence author.

## Ethics Statement

The studies involving human participants were reviewed and approved by the Ethics Administration Office of the West China Hospital, Sichuan University. Written informed consent to participate in this study was provided by the participants’ legal guardian/next of kin.

## Author Contributions

TZ and XM are responsible for research design and project management. HX is responsible for data collection. YZ and XL are responsible for image processing and feature extraction. CC is responsible for article writing. YL and XZ are responsible for statistical analysis. All authors contributed to the article and approved the submitted version.

## Conflict of Interest

The authors declare that the research was conducted in the absence of any commercial or financial relationships that could be construed as a potential conflict of interest.

## Abbreviations

PNETs, Pancreatic Neuroendocrine Tumors; CT, Computed Tomography; MRI, Magnetic Resonance Imaging; PET, Positron Emission Computed Tomography; OS, Overall Survival; ROC, Receiver Operating Characteristic; ROI, Region Of Interest; AUC, Area Under the Curve; GLCM, Gray-Level Co-Occurrence Matrix; GLRLM, Gray Level Run Length Matrix; SRE, Short Run Emphasis; LRE, Long Run Emphasis; LGRE, Low Grey Level Run Emphasis; HGRE, High Gray Level Run Emphasis; SRLGE, Short-Run Low Grey Level Emphasis; SRHGE, Short-Run High Grey Level Emphasis; LRLGE, Long-Run Low Grey Level Emphasis; LRHGE, Long-Run High Grey Level Emphasis; GLNU, Grey Level Non-Uniformity; RLNU, Run Length Non-Uniformity; RP, Run Percentage; NGLDM, Neighborhood Grey Level Difference Matrix; GLZLM, Gray Level Zone Length Matrix; SZE, Short Zone Emphasis; LZE, Long Zone Emphasis; LGZE, Low Gray Level Zone Emphasis; HGZE, High Grey Level Zone Emphasis; SZLGE, Short Zone Low Grey Level Emphasis; SZHGE, Short Zone High Grey Level Emphasis; LZLGE, Long Zone Low Grey Level Emphasis; LZHGE, Long Zone High Grey Level Emphasis; ZLNU, Zone Length Non-Uniformity; ZP, Zone Percentage. DC, Distance Correlation; RF, Random Forest; LASSO, Least absolute shrinkage and selection operator; Xgboost, eXtreme Gradient Boosting; GBDT, Gradient Boosting Decision Tree; LDA, linear discriminant analysis; SVM, Support Vector Machines; AdaBoost, Adaptive Boosting; KNN, K-nearest neighborhood; GaussianNB, Gaussian Naive Bayes; LR, Logistic Regression; DT, Decision Tree.

## Glossary

**Table:**

PNETs	Pancreatic Neuroendocrine Tumors
CT	Computed Tomography
MRI	Magnetic Resonance Imaging
PET	Positron Emission Computed Tomography
OS	Overall Survival
ROC	Receiver Operating Characteristic
ROI	Region Of Interest
AUC	Area Under the Curve
GLCM	Gray-Level Co-Occurrence Matrix
GLRLM	Gray Level Run Length Matrix
SRE	Short Run Emphasis
LRE	Long Run Emphasis
LGRE	Low Grey Level Run Emphasis
HGRE	High Gray Level Run Emphasis
SRLGE	Short-Run Low Grey Level Emphasis
SRHGE	Short-Run High Grey Level Emphasis
LRLGE	Long-Run Low Grey Level Emphasis
LRHGE	Long-Run High Grey Level Emphasis
GLNU	Grey Level Non-Uniformity
RLNU	Run Length Non-Uniformity
RP	Run Percentage
NGLDM	Neighborhood Grey Level Difference Matrix
GLZLM	Gray Level Zone Length Matrix
SZE	Short Zone Emphasis
LZE	Long Zone Emphasis
LGZE	Low Gray Level Zone Emphasis
HGZE	High Grey Level Zone Emphasis
SZLGE	Short Zone Low Grey Level Emphasis
SZHGE	Short Zone High Grey Level Emphasis
LZLGE	Long Zone Low Grey Level Emphasis
LZHGE	Long Zone High Grey Level Emphasis
ZLNU	Zone Length Non-Uniformity
ZP	Zone Percentage
DC	Distance Correlation
RF	Random Forest
LASSO	Least absolute shrinkage and selection operator
Xgboost	eXtreme Gradient Boosting
GBDT	Gradient Boosting Decision Tree
LDA	linear discriminant analysis
SVM	Support Vector Machines
AdaBoost	Adaptive Boosting
KNN	K-nearest neighborhood
GaussianNB	Gaussian Naive Bayes
LR	Logistic Regression
DT	Decision Tree

## References

[1] Dasari A Shen C Halperin D Zhao B Zhou S Xu Y Trends in the Incidence, Prevalence, and Survival Outcomes in Patients With Neuroendocrine Tumors in the United States. JAMA Oncol (2017) 3(10):1335–42. 10.1001/jamaoncol.2017.0589 PMC5824320 28448665

[2] Garcia-Carbonero R Sorbye H Baudin E Raymond E Wiedenmann B Niederle B ENETS Consensus Guidelines for High-Grade Gastroenteropancreatic Neuroendocrine Tumors and Neuroendocrine Carcinomas. Neuroendocrinology (2016) 103(2):186–94. 10.1159/000443172 26731334

[3] Fang JM Shi J A Clinicopathologic and Molecular Update of Pancreatic Neuroendocrine Neoplasms With a Focus on the New World Health Organization Classification. Arch Pathol Lab Med (2019) 143(11):1317–26. 10.5858/arpa.2019-0338-RA PMC7141760 31509453

[4] Kim JY Hong SM Ro JY Recent updates on grading and classification of neuroendocrine tumors. Ann Diagn Pathol (2017) 29:11–6. 10.1016/j.anndiagpath.2017.04.005 28807335

[5] Orditura M Petrillo A Ventriglia J Diana A Laterza MM Fabozzi A Pancreatic neuroendocrine tumors: Nosography, management and treatment. Int J Surg (2016) 28(Suppl 1):S156–62. 10.1016/j.ijsu.2015.12.052 26708853

[6] Akirov A Larouche V Alshehri S Asa SL Ezzat S Treatment Options for Pancreatic Neuroendocrine Tumors. Cancers (Basel) (2019) 11(6):828. 10.3390/cancers11060828 PMC6628351 31207914

[7] Ito T Igarashi H Jensen RT Pancreatic neuroendocrine tumors: clinical features, diagnosis and medical treatment: advances. Best Pract Res Clin Gastroenterol (2012) 26(6):737–53. 10.1016/j.bpg.2012.12.003 PMC3627221 23582916

[8] Lee YJ Lee JM Lee JS Lee HY Park BH Kim YH Hepatocellular carcinoma: diagnostic performance of multidetector CT and MR imaging-a systematic review and meta-analysis. Radiology (2015) 275(1):97–109. 10.1148/radiol.14140690 25559230

[9] Somers I Bipat S Contrast-enhanced CT in determining resectability in patients with pancreatic carcinoma: a meta-analysis of the positive predictive values of CT. Eur Radiol (2017) 27(8):3408–35. 10.1007/s00330-016-4708-5 PMC5491588 28093626

[10] Gonoi W Hayashi TY Okuma H Akahane M Nakai Y Mizuno S Development of pancreatic cancer is predictable well in advance using contrast-enhanced CT: a case-cohort study. Eur Radiol (2017) 27(12):4941–50. 10.1007/s00330-017-4895-8 28631079

[11] Chaddad A Niazi T Probst S Bladou F Anidjar M Bahoric B Predicting Gleason Score of Prostate Cancer Patients Using Radiomic Analysis. (2018) 8:630. 10.3389/fonc.2018.00630 PMC6305278 30619764

[12] Chaddad A Daniel P Niazi T Radiomics Evaluation of Histological Heterogeneity Using Multiscale Textures Derived From 3D Wavelet Transformation of Multispectral Images. (2018) 8:96. 10.3389/fonc.2018.00096 PMC5893871 29670857

[13] Chaddad A Kucharczyk MJ Daniel P Sabri S Jean-Claude BJ Niazi T Radiomics in Glioblastoma: Current Status and Challenges Facing Clinical Implementation. Front Oncol (2019) 9:374. 10.3389/fonc.2019.00374 PMC6536622 31165039

[14] Lubner MG Smith AD Sandrasegaran K Sahani DV Pickhardt PJ CT Texture Analysis: Definitions, Applications, Biologic Correlates, and Challenges. Radiographics (2017) 37(5):1483–503. 10.1148/rg.2017170056 28898189

[15] Di Cataldo S Ficarra E Mining textural knowledge in biological images: Applications, methods and trends. Comput Struct Biotechnol J (2017) 15:56–67. 10.1016/j.csbj.2016.11.002 27994798 PMC5155047

[16] Miles KA Ganeshan B Hayball MP CT texture analysis using the filtration-histogram method: what do the measurements mean? Cancer Imaging (2013) 13(3):400–6. 10.1102/1470-7330.2013.9045 PMC3781643 24061266

[17] Lee SJ Zea R Kim DH Lubner MG Deming DA Pickhardt PJ CT texture features of liver parenchyma for predicting development of metastatic disease and overall survival in patients with colorectal cancer. Eur Radiol (2018) 28(4):1520–8. 10.1007/s00330-017-5111-6 PMC7713793 29164382

[18] Tabari A Torriani M Miller KK Klibanski A Kalra MK Bredella MA Anorexia Nervosa: Analysis of Trabecular Texture with CT. Radiology (2017) 283(1):178–85. 10.1148/radiol.2016160970 PMC5375622 27797678

[19] Canellas R Burk KS Parakh A Sahani DV Prediction of Pancreatic Neuroendocrine Tumor Grade Based on CT Features and Texture Analysis. AJR Am J Roentgenol (2018) 210(2):341–6. 10.2214/AJR.17.18417 29140113

[20] Guo C Zhuge X Wang Q Xiao W Wang Z Wang Z The differentiation of pancreatic neuroendocrine carcinoma from pancreatic ductal adenocarcinoma: the values of CT imaging features and texture analysis. Cancer Imaging (2018) 18(1):37. 10.1186/s40644-018-0170-8 30333055 PMC6192319

[21] Guo C Zhuge X Wang Z Wang Q Sun K Feng Z Textural analysis on contrast-enhanced CT in pancreatic neuroendocrine neoplasms: association with WHO grade. Abdom Radiol (NY) (2019) 44(2):576–85. 10.1007/s00261-018-1763-1 30182253

[22] Choi TW Kim JH Yu MH Park SJ Han JK Pancreatic neuroendocrine tumor: prediction of the tumor grade using CT findings and computerized texture analysis. Acta Radiol (2018) 59(4):383–92. 10.1177/0284185117725367 28766979

[23] D’Onofrio M Ciaravino V Cardobi N De Robertis R Cingarlini S Landoni L CT Enhancement and 3D Texture Analysis of Pancreatic Neuroendocrine Neoplasms. Sci Rep (2019) 9(1):2176. 10.1038/s41598-018-38459-6 30778137 PMC6379382

[24] Ahn SY Park CM Park SJ Kim HJ Song C Lee SM Prognostic value of computed tomography texture features in non-small cell lung cancers treated with definitive concomitant chemoradiotherapy. Invest Radiol (2015) 50(10):719–25. 10.1097/RLI.0000000000000174 26020832

[25] Zhou X Luo Y Peng YL Cai W Lu Q Lin L Hepatic perfusion disorder associated with focal liver lesions: contrast-enhanced US patterns–correlation study with contrast-enhanced CT. Radiology (2011) 260(1):274–81. 10.1148/radiol.11101454 21467250

[26] Zhao YJ Chen WX Wu DS Zhang WY Zheng LR Differentiation of mass-forming intrahepatic cholangiocarcinoma from poorly differentiated hepatocellular carcinoma: based on the multivariate analysis of contrast-enhanced computed tomography findings. Abdom Radiol (NY) (2016) 41(5):978–89. 10.1007/s00261-015-0629-z 27193795

[27] Nioche C Orlhac F Boughdad S Reuze S Goya-Outi J Robert C LIFEx: A Freeware for Radiomic Feature Calculation in Multimodality Imaging to Accelerate Advances in the Characterization of Tumor Heterogeneity. Cancer Res (2018) 78(16):4786–9. 10.1158/0008-5472.CAN-18-0125 29959149

[28] Nardone V Tini P Pastina P Botta C Reginelli A Carbone SF Radiomics predicts survival of patients with advanced non-small cell lung cancer undergoing PD-1 blockade using Nivolumab. Oncol Lett (2020) 19(2):1559–66. 10.3892/ol.2019.11220 PMC6956423 31966081

[29] Chen C Guo X Wang J Guo W Ma X Xu J The Diagnostic Value of Radiomics-Based Machine Learning in Predicting the Grade of Meningiomas Using Conventional Magnetic Resonance Imaging: A Preliminary Study. Front Oncol (2019) 9:1338. 10.3389/fonc.2019.01338 31867272 PMC6908490

[30] van der Schaaf A Xu CJ van Luijk P Van’t Veld AA Langendijk JA Schilstra C Multivariate modeling of complications with data driven variable selection: guarding against overfitting and effects of data set size. Radiother Oncol (2012) 105(1):115–21. 10.1016/j.radonc.2011.12.006 22264894

[31] Kim JH Eun HW Kim YJ Lee JM Han JK Choi BI Pancreatic neuroendocrine tumour (PNET): Staging accuracy of MDCT and its diagnostic performance for the differentiation of PNET with uncommon CT findings from pancreatic adenocarcinoma. Eur Radiol (2016) 26(5):1338–47. 10.1007/s00330-015-3941-7 26253257

[32] Zhu L Dai MH Wang ST Jin ZY Wang Q Denecke T Multiple solid pancreatic lesions: Prevalence and features of non-malignancies on dynamic enhanced CT. Eur J Radiol (2018) 105:8–14. 10.1016/j.ejrad.2018.05.016 30017302

[33] Gazi PM Aminololama-Shakeri S Yang K Boone JM Temporal subtraction contrast-enhanced dedicated breast CT. Phys Med Biol (2016) 61(17):6322–46. 10.1088/0031-9155/61/17/6322 PMC5056786 27494376

[34] Liu X Huang H Yu J Cao G Feng L Xu Q Warfarin compared with aspirin for older Chinese patients with stable coronary heart diseases and atrial fibrillation complications. Int J Clin Pharmacol Ther (2014) 52(6):454–9. 10.5414/CP201996 24755126

[35] Ma X Ling W Xia F Zhang Y Zhu C He J Application of Contrast-Enhanced Ultrasound (CEUS) in Lymphomatous Lymph Nodes: A Comparison between PET/CT and Contrast-Enhanced CT. Contrast Media Mol Imaging (2019) 2019:5709698–. 10.1155/2019/5709698 PMC6364116 30809108

[36] Giganti F Antunes S Salerno A Ambrosi A Marra P Nicoletti R Gastric cancer: texture analysis from multidetector computed tomography as a potential preoperative prognostic biomarker. Eur Radiol (2017) 27(5):1831–9. 10.1007/s00330-016-4540-y 27553932

[37] Gourtsoyianni S Doumou G Prezzi D Taylor B Stirling JJ Taylor NJ Primary Rectal Cancer: Repeatability of Global and Local-Regional MR Imaging Texture Features. Radiology (2017) 284(2):552–61. 10.1148/radiol.2017161375 PMC6150741 28481194

[38] Nelson DA Tan TT Rabson AB Anderson D Degenhardt K White E Hypoxia and defective apoptosis drive genomic instability and tumorigenesis. Genes Dev (2004) 18(17):2095–107. 10.1101/gad.1204904 PMC515288 15314031

[39] Ganeshan B Miles KA Quantifying tumour heterogeneity with CT. Cancer Imaging (2013) 13:140–9. 10.1102/1470-7330.2013.0015 PMC3613789 23545171

[40] van der Pol CB Lee S Tsai S Larocque N Alayed A Williams P Differentiation of pancreatic neuroendocrine tumors from pancreas renal cel carcinoma metastases on CT using qualitative and quantitative features. Abdom Radiol (NY) (2019) 44(3):992–9. 10.1007/s00261-018-01889-x 30603880

[41] Guo C Chen X Xiao W Wang Q Sun K Wang Z Pancreatic neuroendocrine neoplasms at magnetic resonance imaging: comparison between grade 3 and grade 1/2 tumors. Onco Targets Ther (2017) 10:1465–74. 10.2147/OTT.S127803 PMC5349505 28331340

[42] Xu H Guo W Cui X Zhuo H Xiao Y Ou X Three-Dimensional Texture Analysis Based on PET/CT Images to Distinguish Hepatocellular Carcinoma and Hepatic Lymphoma. Front Oncol (2019) 9:844. 10.3389/fonc.2019.00844 31552173 PMC6733884

[43] Suh HB Choi YS Bae S Ahn SS Chang JH Kang SG Primary central nervous system lymphoma and atypical glioblastoma: Differentiation using radiomics approach. Eur Radiol (2018) 28(9):3832–9. 10.1007/s00330-018-5368-4 29626238

[44] Tian Z Chen C Fan Y Ou X Wang J Ma X Glioblastoma and Anaplastic Astrocytoma: Differentiation Using MRI Texture Analysis. Front Oncol (2019) 9:876. 10.3389/fonc.2019.00876 31552189 PMC6743014

[45] Li W Yin Y Quan X Zhang H Gene Expression Value Prediction Based on XGBoost Algorithm. Front Genet (2019) 10:1077. 10.3389/fgene.2019.01077 31781160 PMC6861218

[46] Li J Lu J Liang P Li A Hu Y Shen Y Differentiation of atypical pancreatic neuroendocrine tumors from pancreatic ductal adenocarcinomas: Using whole-tumor CT texture analysis as quantitative biomarkers. Cancer Med (2018) 7(10):4924–31. 10.1002/cam4.1746 PMC6198241 30151864

[47] Fan Y Chen C Zhao F Tian Z Wang J Ma X Radiomics-Based Machine Learning Technology Enables Better Differentiation Between Glioblastoma and Anaplastic Oligodendroglioma. Front Oncol (2019) 9:1164. 10.3389/fonc.2019.01164 31750250 PMC6848260

