# Supplementary material for: Application of Radiomics Analysis Based on CT Combined With Machine Learning in Diagnostic of Pancreatic Neuroendocrine Tumors Patient’s Pathological Grades
Source: Front Oncol. 2021 Feb 11;10:521831. doi: 10.3389/fonc.2020.521831 (PMC7905094; doi:10.3389/fonc.2020.521831)
Supplement: Supplementary file 1 [file DataSheet_1.docx]

**Supplementary material 1 Diagnostic AUC of all models in training and validation set**

|  | **G1 vs G2** | | **G2 vs G3** | | **G1 vs G3** | |
| --- | --- | --- | --- | --- | --- | --- |
| Selection+Classifiers | Training set | Validation set | Training set | Validation set | Training set | Validation set |
| DC+LDA | 0.86 | 0.73 | 0.84 | 0.61 | 0.89 | 0.78 |
| DC+SVM | 0.37* | 0.32* | 0.79 | 0.68 | 0.78 | 0.75 |
| DC+RF | 0.94 | 0.71 | 0.92 | 0.67 | 0.96 | 0.81 |
| **DC+AdaBoost** | **1.00** | **0.82** | **1.00** | **0.70** | **1.00** | **0.85** |
| DC+KNN | 0.90 | 0.81 | 0.86 | 0.69 | 0.92 | 0.83 |
| DC+GaussianNB | 0.81 | 0.77 | 0.83 | 0.62 | 0.90 | 0.81 |
| DC+LR | 0.85 | 0.82 | 0.80 | 0.66 | 0.87 | 0.82 |
| **DC+GBDT** | 1.00 | 0.75 | 1.00 | 0.73 | 1.00 | 0.82 |
| DC+DT | 1.00# | 0.60# | 1.00# | 0.54# | 1.00 | 0.77 |
| RF+LDA | 0.85 | 0.63 | 0.83# | 0.49# | 0.88 | 0.71 |
| RF+SVM | 0.00* | 0.50* | 0.00* | 0.50* | 0.00* | 0.50* |
| RF+RF | 0.98 | 0.72 | 0.92# | 0.59# | 0.97 | 0.76 |
| RF+AdaBoost | 1.00 | 0.89 | 1.00# | 0.63# | 1.00 | 0.76 |
| RF+KNN | 0.80 | 0.59 | 0.76 | 0.48 | 0.81 | 0.62 |
| RF+GaussianNB | 0.68 | 0.65 | 0.65 | 0.60 | 0.50* | 0.42* |
| RF+LR | 0.71 | 0.70 | 0.65 | 0.67 | 0.64 | 0.61 |
| RF+GBDT | 1.00 | 0.80 | 1.00# | 0.65# | 1.00 | 0.65 |
| RF+DT | 1.00# | 0.70# | 1.00 | 0.62 | 1.00 | 0.62 |
| LASSO+LDA | 0.91 | 0.61 | 0.91# | 0.54# | 0.96 | 0.85 |
| LASSO+SVM | 0.21* | 0.29* | 0.24* | 0.39* | 0.18* | 0.24* |
| LASSO+RF | 0.92 | 0.64 | 0.93 | 0.63 | 0.95 | 0.73 |
| LASSO+AdaBoost | 1.00# | 0.53# | 1.00# | 0.52# | 1.00 | 0.82 |
| LASSO+KNN | 0.83 | 0.67 | 0.85 | 0.70 | 0.89 | 0.79 |
| LASSO+GaussianNB | 0.79 | 0.69 | 0.79 | 0.59 | 0.87 | 0.73 |
| LASSO+LR | 0.81 | 0.71 | 0.81 | 0.59 | 0.85 | 0.78 |
| LASSO+GBDT | 1.00# | 0.49# | 1.00# | 0.56# | 1.00 | 0.75 |
| LASSO+DT | 1.00# | 0.57# | 1.00# | 0.59# | 1.00# | 0.68# |
| **Xgboost+LDA** | 0.77 | 0.72 | 0.84 | 0.71 | 0.87 | 0.81 |
| Xgboost+SVM | 0.79# | 0.47# | 0.00* | 0.50* | 0.00* | 0.51* |
| Xgboost+RF | 0.98 | 0.78 | 0.91 | 0.57 | 0.98 | 0.86 |
| Xgboost+AdaBoost | 1.00 | 0.95 | 1.00# | 0.52# | 1.00 | 0.90 |
| Xgboost+KNN | 0.78 | 0.67 | 0.74 | 0.49 | 0.91 | 0.81 |
| Xgboost+GaussianNB | 0.74 | 0.68 | 0.69 | 0.62 | 0.87 | 0.74 |
| Xgboost+LR | 0.77 | 0.81 | 0.67 | 0.60 | 0.78 | 0.70 |
| Xgboost+GBDT | 1.00 | 0.93 | 1.00# | 0.58# | 1.00 | 0.88 |
| Xgboost+DT | 1.00 | 0.80 | 1.00 | 0.58 | 1.00 | 0.80 |
| GBDT+LDA | 0.87 | 0.80 | 0.89 | 0.55 | 0.84 | 0.76 |
| GBDT+SVM | 0.00* | 0.50* | 0.00* | 0.50* | 0.00* | 0.50* |
| GBDT+RF | 0.98 | 0.83 | 0.94 | 0.69# | 0.99 | 0.87 |
| GBDT+AdaBoost | 1.00 | 0.90 | 1.00# | 0.62# | 1.00 | 0.90 |
| GBDT+KNN | 0.81 | 0.64 | 0.80 | 0.63 | 0.81 | 0.62 |
| GBDT+GaussianNB | 0.80 | 0.70 | 0.82 | 0.62 | 0.40 | 0.36 |
| GBDT+LR | 0.86 | 0.75 | 0.83 | 0.57 | 0.62 | 0.59 |
| GBDT+GBDT | 1.00 | 0.89 | 1.00# | 0.66# | 1.00 | 0.81 |
| GBDT+DT | 1.00 | 0.81 | 1.00 | 0.59 | 1.00 | 0.71 |

**Abbreviations:** DC, Distance Correlation; RF, Random Forest; LASSO, Least absolute shrinkage and selection operator; Xgboost, eXtreme Gradient Boosting; GBDT, Gradient Boosting Decision Tree; LDA, linear discriminant analysis; SVM, Support Vector Machines; AdaBoost, Adaptive Boosting; KNN, K-nearest neighborhood; GaussianNB, Gaussian Naive Bayes; LR, Logistic Regression; DT, Decision Tree.

**Note:** * means that the model showed under-fitting. # means that the model showed over-fitting. All AUC values in the table are the mean values of AUC values obtained from 10 cross validation.
